# The nuclear lamina is a hub for the nuclear function of Jacob

**DOI:** 10.1186/s13041-020-00722-1

**Published:** 2021-01-12

**Authors:** Sebastian Samer, Rajeev Raman, Gregor Laube, Michael R. Kreutz, Anna Karpova

**Affiliations:** 1 RG Neuroplasticity, Leibniz Institute for Neurobiology, 39118 Magdeburg, Germany; 2 Center for Behavioral Brain Sciences, Otto Von Guericke University, 39106 Magdeburg, Germany; 3 Leibniz Group ‘Dendritic Organelles and Synaptic Function’, Center for Molecular Neurobiology, ZMNH, University Medical Center Hamburg-Eppendorf, 20251 Hamburg, Germany

**Keywords:** Jacob/Nsmf, Lamin B1, CRM1, Nuclear export, NMDAR, Synapse-to-nucleus

## Abstract

Jacob is a synapto-nuclear messenger protein that couples NMDAR activity to CREB-dependent gene expression. In this study, we investigated the nuclear distribution of Jacob and report a prominent targeting to the nuclear envelope that requires NMDAR activity and nuclear import. Immunogold electron microscopy and proximity ligation assay combined with STED imaging revealed preferential association of Jacob with the inner nuclear membrane where it directly binds to LaminB1, an intermediate filament and core component of the inner nuclear membrane (INM). The association with the INM is transient; it involves a functional nuclear export signal in Jacob and a canonical CRM1-RanGTP-dependent export mechanism that defines the residing time of the protein at the INM. Taken together, the data suggest a stepwise redistribution of Jacob within the nucleus following nuclear import and prior to nuclear export.

## Introduction

The nuclear envelope consists of the outer and inner nuclear membrane (INM) [1, 2]. Both membranes are segregated in functional and structural terms. While the outer nuclear membrane (ONM) is an extension of the endoplasmic reticulum (ER) and is continuous with the ER in dendrites and a subset of spines (3–5), the INM is physically separated and serves, among other functions, as a Ca^2+^-source for nuclear Ca^2+^-waves that switch on gene expression required for learning and memory and adaptive responses [3–5]. In addition, the INM can regulate gene transcription, leading to silencing through the association with heterochromatin [6–9] and scavenging of active signaling molecules involved in gene regulation [10, 11]. These functional properties come along with a distinct membrane composition. The INM is tightly associated with the nuclear lamina, a meshwork that is composed to a large degree of lamins, which are intermediate filaments that have been shown to contribute to invaginations of the inner nuclear membrane, sequestration of transcriptional regulators [3, 5] and organization of chromatin into lamina-associated domains [12, 13].

Protein transport from synapse to nucleus is an established means for the regulation of gene expression [14, 15]. Several proteins localized at glutamatergic synapses translocate in an activity-dependent manner to the nucleus [14–19] where they control different transcriptional programs involved in the modulation of synaptic transmission and structural plasticity. In many cases their nuclear translocation is driven by the activation of *N*-methyl-d-aspartate glutamate receptors (NMDAR) and require an importin-mediated active retrograde transport along microtubules. One of these messengers is Jacob, a protein that encodes and transduces the synaptic and extrasynaptic origin of GluN2B NMDAR signals to the nucleus [15]. In the nucleus, Jacob docks a signalosome to the cAMP response element binding protein (CREB)-complex [20], however, little is known about its subnuclear localization and corresponding dynamics. In previous work employing immunocytochemical staining and confocal imaging, we observed that Jacob localizes to the nuclear rim in hippocampal neurons [14, 16, 21, 22]. This observation raised a number of questions. An association with the outer nuclear membrane could indicate a local accumulation following long-distance transport along the ER, while an association with the inner nuclear membrane would point to a distinct expression of Jacob in subnuclear compartments with potential functional implications.

## Methods

### Animals and tissue collection for EM

Wistar rats were bred and housed in the animal facility of the Leibniz Institute for Neurobiology, Magdeburg. Experiments were conducted following ethical animal research standards defined by the German Law/European directive and approved by the Landesverwaltungsamt Saxony-Anhalt (Referat 203, Verbraucherschutz und Veterinärangelegenheiten). The competent authority follows the advice of an official animal welfare committee of the Federal State of Saxony-Anhalt, Germany.

For EM experiments, male adult Wistar rats were deeply anaesthetized using a mixture of Ketavet (Parke-Davis) and Domitor (Pfizer) and then transcardially perfused with a solution containing 0.9% NaCl for 1 min followed by 4% paraformaldehyde (PFA) and 0.05 glutaraldehyde in 0.1 M PBS for 20 min. Brains were removed and post-fixed in 4% PFA solution for 20 h. For pre-embedding immunogold labeling, brains were rinsed in 0.1 M PB and freeze-protected with 1 M sucrose in 0.1 M PB. Tissue was frozen at − 40 °C in iso-pentane and 25 µm frontal sections were prepared. For cryo-immunogold labeling, brains were rinsed in 0.1 M PB and sliced at 700 µm using a vibratome. Brain tissue was freeze-protected with 2.3 M sucrose. Samples of cerebral cortex (1 × 2 mm) were mounted on cryo holders and frozen by immersion in liquid nitrogen.

### Pre-embedding immunogold labeling for EM

For immunoelectron microscopy, freely floating sections were rinsed in 0.01 mol/L phosphate buffered saline (PBS) at pH 7.4, treated for 15 min with 1% sodium borohydride in PBS and thoroughly washed in PBS. Thereafter, sections were pre-treated for 30 min in a blocking and permeabilizing solution consisting of 10% normal goat serum (NGS; Interchem, Bad Kreuznach, Germany), 0.1% saponine, in PBS at 22 °C. Rabbit anti-Jacob antibody (1:1000) was applied for 36 h in PBS containing 10% NGS, 0.1% saponine and 0.1% sodium azide at 4 °C. Sections were then thoroughly rinsed in PBS, pre-treated for 1 h with 0.1% acetylated BSA (Aurion, Wageningen, the Netherlands) and 0.1% Tween 20 in PBS and exposed for another 24 h to the secondary antibody, 1 nm colloidal gold-coupled goat anti-rabbit IgG (Auroprobe; Amersham, Little Chalfont, UK) diluted 1:50 in the same solution. After several PBS washing steps for at least 2 h, sections were post-fixed for 15 min with 2% glutaraldehyde at 22 °C and then thoroughly rinsed in 150 mmol/L aqueous sodium nitrate. Gold particles with a diameter of 1 nm were silver enhanced for 1–1.5 h using a commercial developer (Intense-M, Amersham) supplemented with one-third (v/v) gum arabic (100 g dissolved in 200 mL H_2_O; Sigma). Development was stopped by several washes in 150 mmol/L aqueous sodium nitrate. After three additional washes in 0.15 mol/L acetate buffer (pH 5.6), silver deposits were stabilized by a modified gold-toning procedure [23]. Sections were treated for 10 min in 0.05% gold chloride (Sigma) in 150 mmol/L acetate buffer, pH 5.5, followed by a 5 min rinse in 150 mmol/L acetate buffer (pH 5.6) at 4 °C. Sections were transferred to 0.1 mol/L cacodylate buffer (CB), post-fixed for 30 min with 1% osmium tetroxide in 0.1 mol/L CB, and washed several times in CB. Finally, sections were dehydrated in a graded series of ethanol for 10 min each, including block staining with 2% uranyl acetate (Serva) in 70% ethanol and then flat embedded in Durcupan ACM (Fluka). Ultrathin sections were prepared with an Ultracut UCT (Leica), contrasted with uranyl acetate and lead citrate and analyzed with a Zeiss EM 900 equipped with a 1 K slow scan CCD camera.

### Immunogold labeling of thin cryosections for electron microscopy

Thin cryosections were prepared and labeled according to a cryo-immunogold technique originally introduced by Tokuyasu [24] and modified by Griffiths [25]. Briefly, thin cryosections of the parietal cerebral cortex were prepared at a thickness of 100 nm. Then, sections were collected on droplets of a mixture of 2.3 M sucrose and 2% methyl cellulose (1:1) and subsequently incubated on droplets of the following solutions: (1) 50 mM glycine in phosphate buffered saline, pH 7.4 (PBS) for 10 min; (2) 10% normal goat serum (NGS) in PBS (15 min); (3) rabbit anti-Jacob antibody (1:100) in PBS containing 10% NGS and 0.1% sodium azide for 20 hs; (4) PBS buffer (3 × 5 min); (5) 0.1% bovine serum albumin (BSA) in PBS (20 min), (6) gold-conjugated anti-rabbit antibody (British Biocell, 1:50) for 4 h; (7) PBS buffer (6 × 3 min); (8) 2.5% glutaraldehyde in PBS (10 min) for postfixation; (9) distilled water (4 × 2 min). Sections were then floated on drops of 1.5% methyl cellulose, containing 2% uranyl acetate (10 min on ice) for contrasting and analyzed with a Zeiss EM 900 equipped with a 1 K slow scan CCD camera.

### Expression constructs and site directed mutagenesis

The following plasmids were obtained from AddGene: pmCherry-RanQ69L (#30309, [26]), hRanT24N-pmCherry (pTK21 #37396, [27]), mTagRFP-hLaminB1 (#58020, [28]), 3xFLAG-hCRM1 (Addgene #17647, [29]). For recombinant protein production, CRM1 and LaminB1 were further subcloned into Intein-pMXB10 vector between NdeI and XhoI restriction sites with the following primers: CRM1-fw 5′-AATTATCATATGCCAGCAATTATGACAATGTTA-3′, CRM1-rev 5′-TCCAGAAGAAATGTGTGATCTCGAGTAATAT-3′, LaminB1-fw 5′-ATTATTCATATGGCGACTGCGACCCCCGT-3′, LaminB1-rev 5′-ATGTGGCTCGAGCATAATTGCACA GCTTCT-3′. WT-Jacob-GFP, Jacob-ΔNLS-GFP, WT-Jacob-Myc, MBP-45–532-Jacob were described previously [14, 15]. Jacob NES mutant constructs (Jacob-L476A-GFP and MBP-45-532-Jacob-L476A) were generated using QuikChenge Site-Directed Mutagenesis (STRATAGENE) with the following primers: mNES-fw 5′ CGGGGAGAAGGCATTCCAGAACC 3′ and mNES-rev 5′ TTAGGGTTCCCCAGGATG 3'.

### Neuronal and HEK293T cell culture and transfection

Rat hippocampal primary neuronal cultures were prepared from E18-E19 as described previously [15] and kept in Neurobasal (Gibco) neuronal medium supplemented with B-27 (Gibco) and 0.5 mM glutamine (Gibco). Neurons were transfected with Jacob mutant constructs at DIV16/17 using lipofectamine2000 (Invitrogen). HEK293T cells cultured in Dulbecco’s Modified Eagle’s Medium (DMEM, Gibco #41966-029) supplemented with 10% Fetal Calf Serum (FBS, Gibco #10270106) and an antibiotic penicillin/streptomycin cocktail were transfected the next day after splitting using the calcium phosphate method.

### Compounds and treatment

Anisomycin (7.5 μM), 4-AP (2.5 mM) and leptomycin-B (LMB; 0.1 nM) were purchased from Sigma, D-APV (20 μM) and ifenprodil (5 μM), bicuculline (50 μM) were purchased from Tocris, and NVP-AAM077 (50 nM) was a kind gift from Dr. Auberson (Novartis). Treatments of hippocampal neurons with D-APV, 4-AP/bicuculline were performed in the presence of anisomycin. Cultures were fixed 30 min after D-APV, 4-AP/bicuculline or leptomycin-B treatment. Ifenprodil and NVP-AAM077 were applied for 28 h and all neurons were immunostained against Jacob and co-stained with anti-MAP2 antibodies. Hoechst or DAPI were used as a nuclear counterstain. Jacob immunoreactivity was measured as mean grey value in arbitrary units of pixel intensity using the Image J software and normalized to untreated conditions.

### Antibodies

Custom-made, affinity-purified antibodies generated against two short amino acid stretches of rat Jacob (285–299 and 400–314, Jb150) have been described previously [14, 21, 22]. The following commercial primary antibodies were used in the present study: mouse monoclonal anti-MAP2 (Sigma-Aldrich, M4403), anti-MAP2, Alexa Fluor 488 conjugated (Merck, MAB 3418x), mouse monoclonal anti-LaminB1 (ProteinTeck, 3C10G12), rabbit polyclonal anti-CRM1 (ProteinTeck), mouse monoclonal anti-MBP (NEB), mouse monoclonal anti-GFP (BioLegend, B34), and rabbit polyclonal anti-tRFP (EVROGEN). For imaging and WB analysis, the following secondary antibodies were used: anti-rabbit, anti-mouse secondary antibodies conjugated with Alexa Fluor 488, 561 or 647 (Life Technologies), peroxidase-conjugated anti-mouse IgG (Dianova) and anti-rabbit IgG (Dianova).

### Immunocytochemistry, proximity ligation assay and confocal microscopy

For immunocytochemical analysis, dissociated neuronal cultures were fixed with 4% PFA solution for 5 min at room temperature and after several washing steps with PBS, cells were permeabilized with 0.1% Triton X-100 in PBS for 10 min. Next, cells were incubated with blocking buffer containing 2% Glycine, 2% BSA, 0.2% gelatin and 50 mM NH_4_Cl for 1 h at RT. Incubation with primary antibodies was done in a blocking buffer at 4 °C overnight. Secondary antibodies were diluted in a blocking buffer 1:500 and applied for 1 h at RT. After washing in PBS, either DAPI or Hoechst was added for 10 min and coverslips were then rinsed in water and mounted with Mowiol 4–88 (Calbiochem/Merck Chemicals Ltd.).

For proximity ligation assay (PLA), neuronal cultures were treated as described above and after incubation with primary antibodies overnight at 4 °C, coverslips were washed with Duolink wash buffer A (Sigma-Aldrich) and incubated with Duolink PLA probes PLUS and MINUS in a humidity chamber for 1 h at 37 °C. For ligation of the probes, coverslips were washed with buffer A and then incubated with ligase for 1 h at 37 °C. For amplification step, coverslips were washed with buffer A and then incubated with polymerase and Duolink FarRed Detection Reagent for 100 min at 37 °C. Coverslips were washed two times with Duolink wash buffer B for 5 min and one time with 0.01 × buffer B for 1 min. Prior to incubation with other antibodies for immunocytochemistry or DAPI counterstaining, coverslips were washed with PBS and afterwards, mounted with Mowiol 4-88.

Images were acquired with a 63× (Leica) objective lens along the z-axis with 300 nm z-step in a 512 × 512 and 1024 × 1024-pixel formats at 8-bit and 16-bit image depth with at least two times frame average at 400 Hz laser frequency using either the SP8 -or SP5 CLSM system (Leica-Microsystems, Mannheim, Germany) equipped with white light laser (WLL). Nuclear Jacob immunoreactivity was measured as the mean grey value in arbitrary units of pixel intensity using Fiji software. For Jacob-LaminB1 co-expression analysis and PLA, raw confocal and STED images were deconvolved using Deconvolution wizard (Huygens Professional, SVI), where we first calculated the theoretical point spread function (PSF) based on optical microscopic parameters provided by original *.lif-files, and subsequently used optimized iteration mode.

STED images were acquired on a SP8 CLSM setup (Leica) using a 100x/1.4 oil objective (HC PL APO CS2, Leica). The STED laser (775 nm) was set to maximum power and used to deplete the white light laser excitations at 561 nm and 633 nm with optimized STED laser delay time. Gating of hybrid detectors was set to 0.5–6 ns. The line profiles from STED images were generated from an average of two optical sections.

### Time-lapse imaging

Hippocampal primary neurons were transfected at DIV9 with the plasmid expressing WT-Jacob-GFP using lipofectamine 2000. Time-lapse imaging was performed as described previously [14, 30]. Z-stacks throughout the nucleus were acquired every 2 min and LMB was applied 10 min after recording as indicated with the timeline in the corresponding figure.

### Heterologous co-immunoprecipitation (Co-IP) assay

HEK-293 T cells were co-transfected via the calcium phosphate precipitation method with the following plasmids: WT-Jacob-Myc and TagRFP-LaminB1 or tagRFP control. 24 h post-transfection cells were harvested and lysed in 1 ml of RIPA buffer containing 50 mM Tris–HCl pH 7.4, 150 mM NaCl and 1% Triton X-100 and 0.1% sodium dodecyl sulfate (SDS), PIC and PhosStop for 1 h at 4 °C. The cleared by centrifugation cell lysate was incubated with anti-Myc antibody-coated magnetic beads (MultiMACS GFP Isolation Kit, Miltenyi Biotec GmbH, Germany) and immunoprecipitation was performed following the manufacturer’s protocol (Mitenyibiotec, Bergisch-Gladbach, Germany) with subsequent WB analysis.

### Induction and purification of fusion proteins for pull-down assay

Proteins were bacterially produced and purified as previously described [31]. In brief, *E. coli* SHuffle (C3026) cells were transformed with either pMAL or LaminB1(PMXB10) vectors and E. coli ExpressLysY/Iq cells were transformed with CRM1 (PMXB10) vector. All bacterial cultures were grown at 30 °C until OD_600_ reaches 0.4–0.8 and subsequently 0.4 mM of IPTG was added for additional 16 °C. For purification of LaminB1 and CRM1, bacterial cell lysates were prepared in 20 mM Tris pH 8.5 500 mM NaCl, sonicated, centrifuged at 20.000*g* and then the supernatant was loaded onto chitin resin and bound to columns. After several washing steps, columns were incubated with the wash buffer containing 50 mM dithiothreitol (DTT) for 24 h at 10 °C. Proteins were eluted and run on the SDS-PAGE gel for the purity checking. For interaction assays between Jacob and LaminB1, and also between Jacob and CRM1, the matrix (amylose-MBP) alone or coupled with Jacob proteins (amylose-MBP-45-532-Jacob and amylose-MBP-45-532-Jacob-L476A) was washed with the buffer containing 20 mM Tris pH 7.5, 1 mM DTT, 3 mM EDTA, 100 mM NaCl, 0.3% TritonX-100, protease inhibitors (Complete, Roche). Subsequently, amylose resin was incubated with 20 μg of an untagged-LaminB1, untagged-CRM1 or with HEK293T cell extract expressing CRM1 overnight at 4 °C and all proteins were eluted and analyzed by WB.

### Quantification and statistical analysis

Data in the manuscript is shown as mean ± S.E.M. Graphs and statistical analysis were made with GraphPad Prism (GraphPad Software). Statistical tests used are indicated in the figure legends. The number of subjects considered for statistical comparison is included in the graphs.

## Results

### The association of Jacob with the nuclear envelope depends upon NMDAR activity

**Fig. 1:**
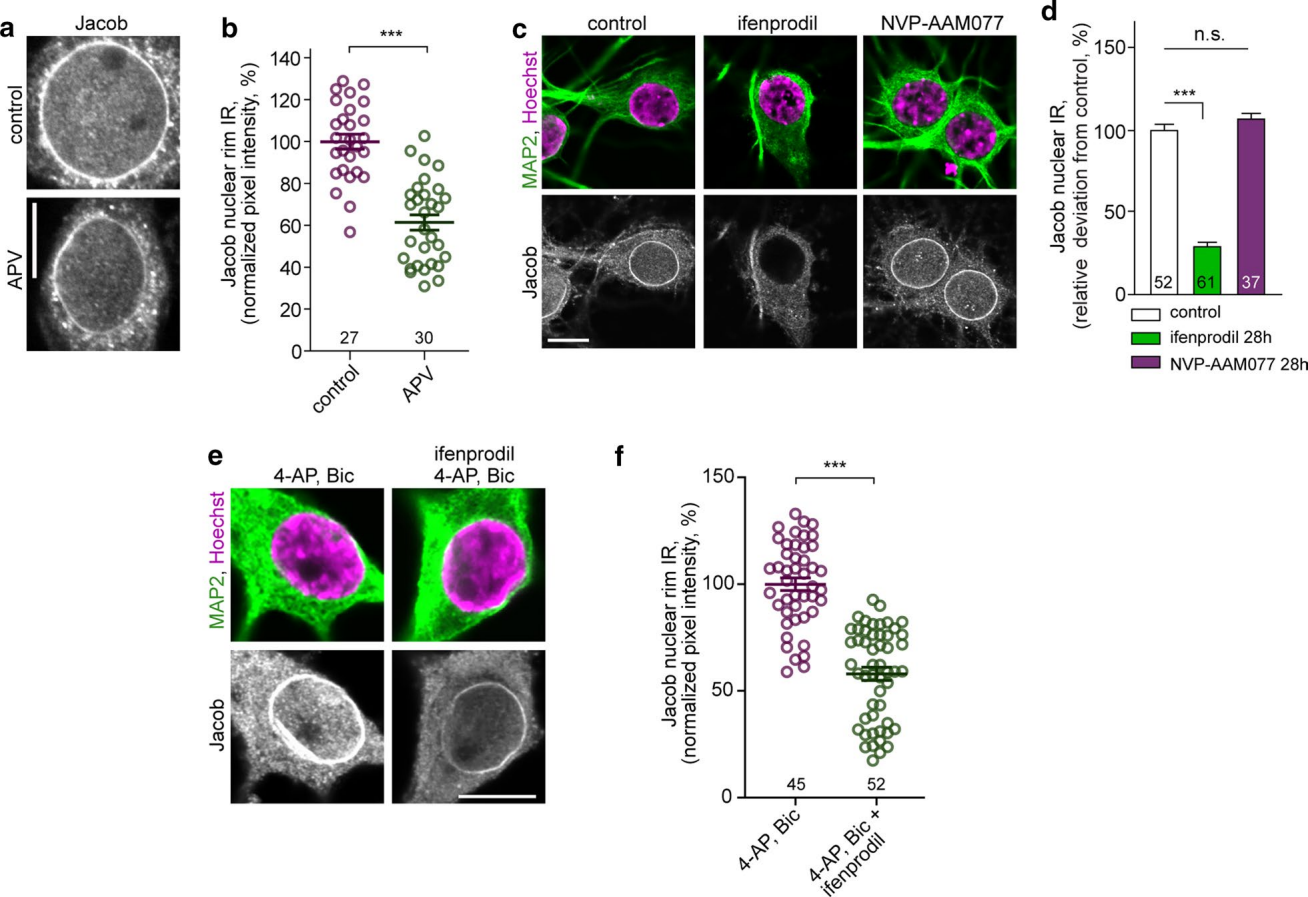
Association of Jacob with the nuclear envelope requires activity of GluN2B but not GluN2A containing NMDAR. **a**, **b** Treatment of hippocampal primary neurons (DIV16/18) with the competitive NMDA receptor antagonist APV (20 µM) for 30 min in the presence of anisomycin results in decreased nuclear envelope association of Jacob. Scale bar indicates 10 µm. **b** Quantification of Jacob IR at the nuclear rim. (***) indicate *p* < 0.001 (ANOVA, Bonferroni). **c**, **d** Chronic treatment of neurons with ifenprodil (5 µM) but not with NVP-AAM077 (50 nM) abolished Jacob nuclear IR as well as association with the nuclear envelope. Depicted are confocal images of hippocampal primary neuron at DIV16/18 immunolabeled with anti-Jacob antibody and co-labeled against MAP2 and stained with DAPI. **d** Quantification of Jacob IR in the nucleus upon the treatment. (***) indicate *p* < 0.001; n.s. = non-significant (ANOVA, Bonferroni). **e**, **f** Treatment of hippocampal primary neurons (DIV16/18) with ifenprodil (5 µM) for 30 min during enhanced synaptic activity prevents accumulation of Jacob at the nuclear envelope. Scale bar indicates 10 µm. **b** Quantification of Jacob IR at the nuclear rim. (***) indicate *p* < 0.001 (Two-tailed t-test)

Protein transport from synapse to nucleus and subsequent nuclear import of Jacob requires NMDAR activity. We therefore first asked whether the association of the protein with the nuclear envelope is regulated by neuronal activity. Immunofluorescence staining of hippocampal primary neurons with a previously characterized antibody [14, 16, 21, 22] revealed a strong association of Jacob with the nuclear rim region (Fig. 1a), indicating that basal activity of neurons in culture is sufficient for the localization at the nuclear envelope. We then set out to verify that the association with the nuclear envelope is regulated by NMDAR activity, and treated hippocampal primary neurons with the NMDAR antagonist APV for 30 min. Interestingly, under these conditions we found a significantly weaker association of Jacob with the nuclear envelope (Fig. 1a, b). Incubation of cultured neurons for longer time periods with an antagonist for NMDAR containing the GluN2B subunit, ifenprodil, resulted in clearly reduced nuclear and nuclear rim staining intensity, while application of the antagonist NVP-AAM077 at concentrations where the compound mainly targets GluN2A-containing NMDAR [32] had no effect (Fig. 1c, d). Next, we preincubated neuronal cultures with ifenprodil and stimulated cells by application of bicuculline and 4-AP for 30 min (Fig. 1e, f). The presence of ifenprodil during enhanced synaptic activity resulted in reduced nuclear accumulation of Jacob [16] and also reduced association of Jacob with the nuclear envelope. Collectively these data suggest that activity of GluN2B-containing NMDAR controls the nuclear rim localization of Jacob.

### Jacob prominently localizes to the inner nuclear membrane

**Fig. 2:**
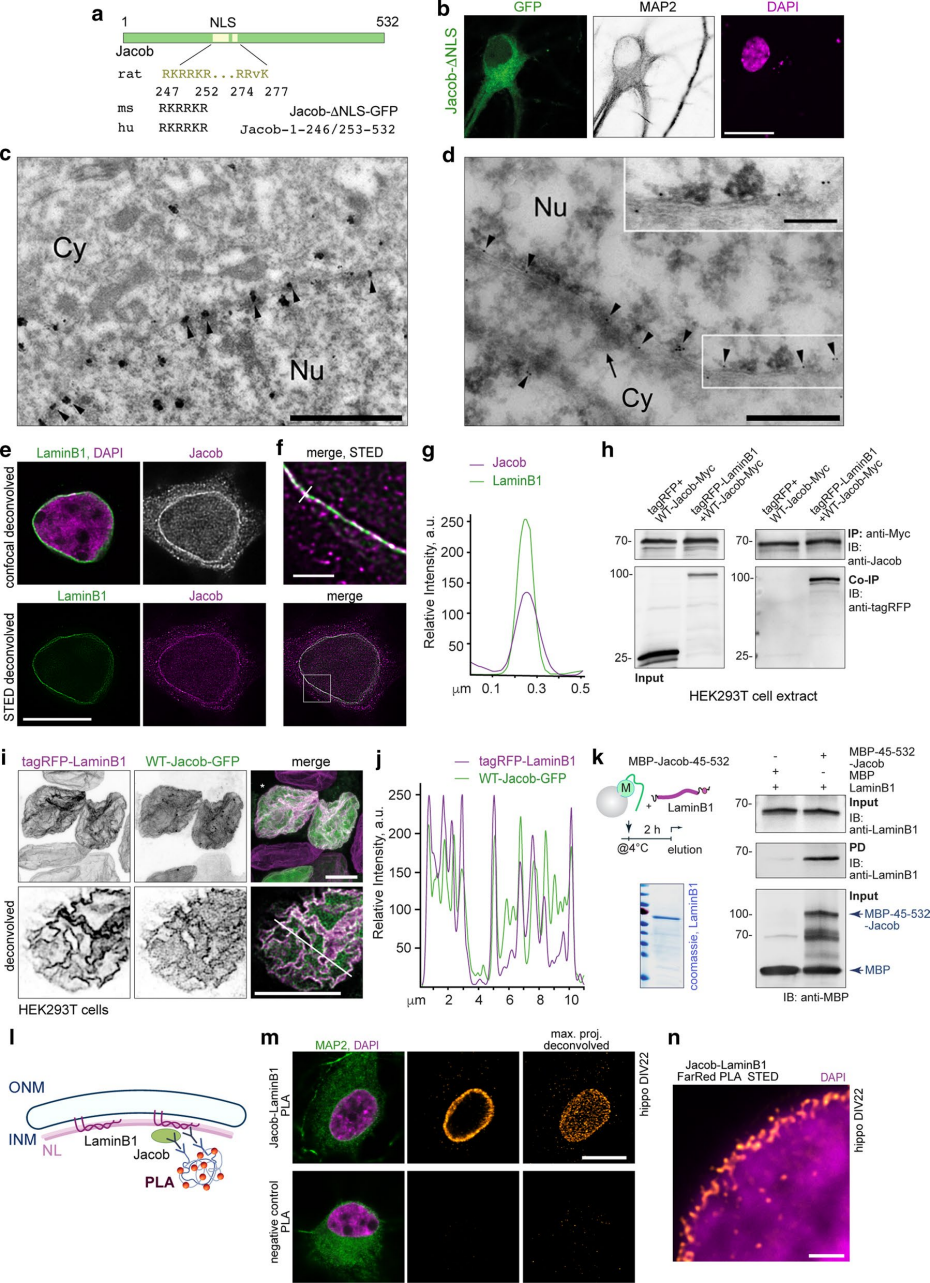
Jacob associates with the inner nuclear envelope via direct binding to LaminB1. **a** Schematic representation of the nuclear localization signal of Jacob and its conservation among species. **b** Jacob lacking the NLS does not associate with the nuclear envelope. Depicted are confocal images of a hippocampal primary neuron (17DIV) expressing ΔNLS-Jacob-GFP and co-labeled with anti-MAP2 and DAPI. **c**, **d**. Immunogold pre-embedding (**c**) and cryosection (**d**) labeling of Jacob in rat cortical pyramidal neurons. **c** Silver-enhanced gold particles of irregular shape were observed predominantly along the inner leaflet (arrowheads) of the nuclear envelope. **d** On thin Tokuyasu cryo-sections, Jacob immunosignal (10 nm gold particles) was observed at the inside of the nuclear envelope (arrowheads). Note the vesicular structure closely associated with the outer nuclear membrane (arrow). The boxed area is shown at increased magnification in the inset. *Cy* cytoplasm, *Nu*  nucleus. Scale bars are: 1 µm in (**c**); 500 nm in (**d**); 200 nm in inset in (**d**). **e**–**g**. STED imaging confirms a tide association of Jacob with laminB1 in hippocampal primary neurons as indicated with the line profile (**g**; line in **f**). Upper panel (**e**) represents Jacob and LaminB1 labeling with confocal resolution; lower panel represents STED images. Box in the merged image indicate the area selected for higher magnification image (**f**). Scale bar in (**e**) indicates 10 µm. Scale bar in (**f**) indicates 1 µm. **h** Co-Immunoprecipitation analysis revealed that Jacob and LaminB1 are part of one protein complex. **i**, **j** WT-Jacob-GFP is recruited to nuclear invaginations which result from tRFP-LaminB1 overexpression, indicated by the line profile. **k** Jacob directly binds LaminB1 as confirmed by a pull-down assay with recombinant proteins. Schematic represents the time-line of the experiment. An intein-tag was used for LaminB1 purification and was cleaved by addition of DTT. Anti-LaminB1 antibodies were used for detection of recombinant protein. **l**–**n** Jacob directly associate with LaminB1 in hippocampal primary neurons as confirmed by proximity ligation assay. **l** Schematic representation of the PLA assay between Jacob and LaminB1. *NL* nuclear lamina. **m** Confocal images of hippocampal primary neurons showing the signal of proximity ligation at the nuclear periphery. Right panel represents deconvolved confocal maximal projection images of nuclear PLA. Scale bar indicates 10 µm. **n** STED image of nuclear proximity ligation between Jacob and LaminB1. Scale bar indicates 1 µm

We have previously shown that Jacob harbours a bipartite nuclear localization sequence (NLS, aa 427-252; 274-277) located within exons 6 and 7 (Fig. 2a) [14, 30]. When we overexpressed Jacob carrying a mutation of the nuclear localization signal fused to GFP, we observed a cytoplasmic localization without accumulation at the nuclear envelope (Fig. 2a, b). To directly address the localization of Jacob at either the ONM or INM, we employed pre-embedding immunogold and cryosection labeling techniques for electron microscopy (EM) in brain sections of cortical pyramidal neurons. Silver-enhanced gold particles after pre-embedding labeling were observed in euchromatic and heterochromatic areas of the nucleoplasm and were also found to be associated with the nuclear envelope (Fig. 2c). Interestingly, they were largely detected inside of the INM (arrowheads). In a complementary approach employing a cryosection post-embedding immunogold labeling technique known for better preservation of membranous structures, we could clearly detect Jacob in association with the nucleoplasmic face of the INM (arrowheads Fig. 2d). Immunogold particles were only occasionally associated with the outer membrane of the nuclear envelope (Fig. 2d).

### Jacob directly binds to LaminB1

Given the close proximity of immunogold particles associated with the INM, we set out to define a molecular mechanism that links Jacob to the INM. We have previously shown that Jacob directly associates with the intermediate filament α-internexin [15] and we therefore reasoned that the association with the INM might be mediated via direct interaction with the nuclear lamins that are immediately adjacent to the INM. LaminB1 is an intermediate filament and a component of the INM lamina facing the nucleoplasm. Together with LaminB2 and LaminA/C, it represents one of the main components of the INM meshwork. Recent studies employing super-resolution imaging [33, 34] revealed that each lamin forms a distinct, but overlapping network [35], in which LaminB1 is more tightly associated with the INM than other lamins [36]. To initially test this idea, we performed STED microscopy in hippocampal primary neurons and found prominent co-localization of Jacob with LaminB1 (Fig. 2e–g). Next, we conducted co-immunoprecipitation experiments with tag-specific antibodies following heterologous expression of both proteins in HEK293T cells under stringent buffer conditions. We indeed detected tagRFP-LaminB1 in one complex with WT-Jacob-Myc when we employed an anti-myc-antibody for immunoprecipitation (Fig. 2h). Confocal imaging and subsequent line profiling show co-recruitment of Jacob into nuclear lamina following ectopic expression (Fig. 2i, j). To confirm a direct interaction, we then performed MBP pull-down assays with bacterially-expressed and purified recombinant proteins (Fig. 2k). For protein stability reasons, we used a truncated version of Jacob-(d44. We found a tight interaction of Jacob fused to MBP and coupled to an amylose resin with untagged LaminB1. In control experiments LaminB1 did not bind to MBP coupled to the matrix (Fig. 2k). Proximity ligation assay followed by STED imaging employing anti-Jacob and anti-LaminB1 antibodies further confirmed the interaction between the two proteins inside the nucleus of primary hippocampal neurons (Fig. 2l–n, Additional file 1: movie S1).

### The recruitment to the INM requires the presence a nuclear export signal in Jacob

**Fig. 3:**
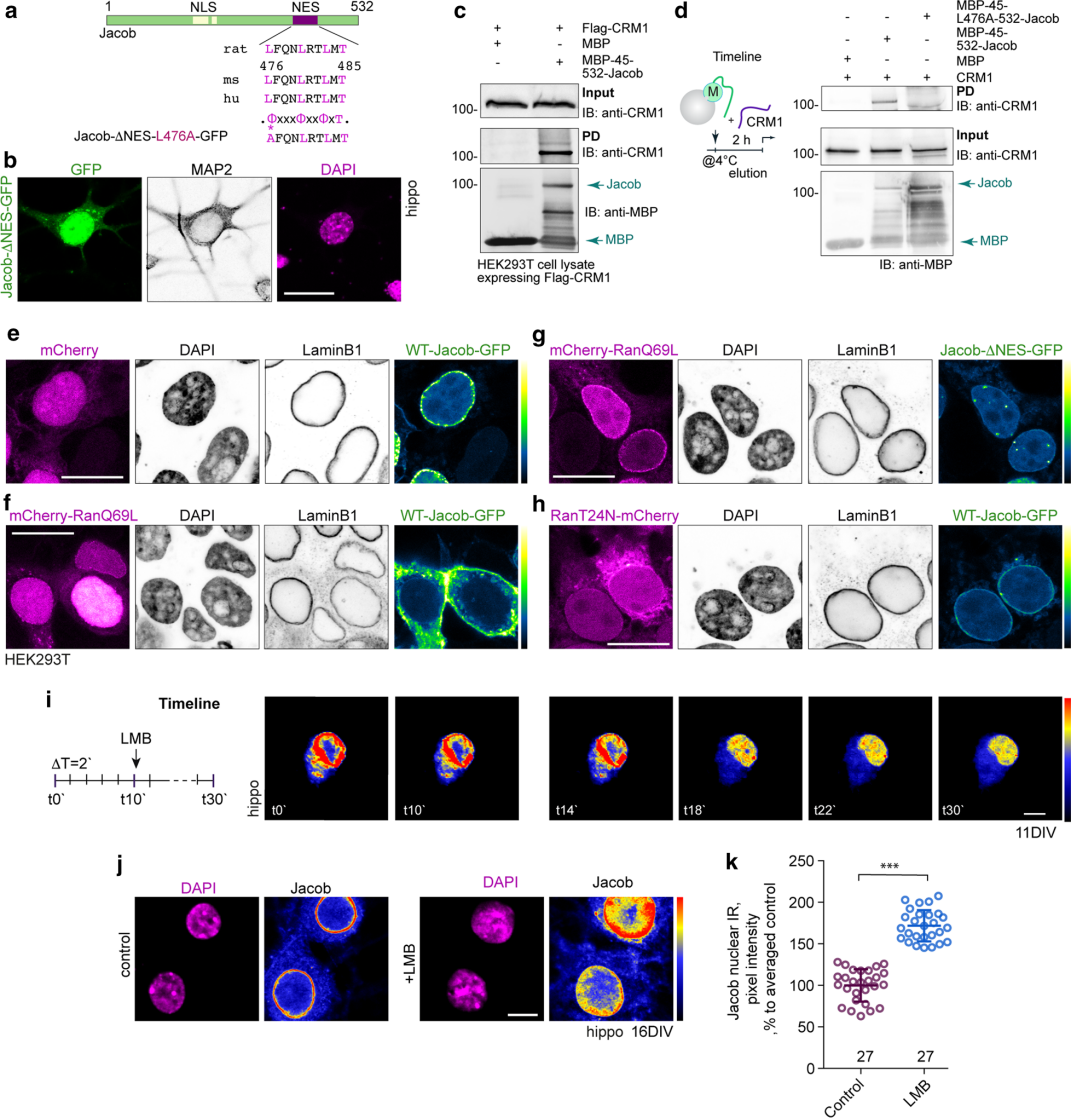
Jacob contains a nuclear export signal that is responsible for its association with the nuclear lamina and Jacob is actively exported from the nucleus via a CRM1- and RanGTP-dependent mechanism. **a** The primary structure of Jacob contains a Class1a CRM1-dependent NES that is conserved across species. A point mutation (L476A) was introduced in the Jacob sequence (indicated by asterisk). **b** A point mutation within the Jacob NES (L476A) altered the subcellular localization of the protein. Depicted are confocal images of a hippocampal neuron (DIV16/18) expressing mNES-Jacob-GFP. The nuclear localization of the mutant protein is altered with no accumulation at the nuclear envelope. Scale bar is 20 μm. **c**, **d** WT-Jacob but not the NES mutant directly associates with CRM1. **c** Pull-down assay performed with bacterially produced recombinant MBP-45-532-Jacob and HEK293T cell extract expressing CRM1. **d** Recombinant NES-Jacob mutant does not bind to recombinant CRM1 as confirmed by a pull-down assay. Schematic represents the timeline of the experiment. **e**–**h** Jacob ectopically expressed in HEK293 cells shows association with the nuclear envelope that is clearly reduced in the presence of GTP-locked form of Ran pmCherry-RanQ69L, but not in the presence of RanGDP-locked mutant (T24N). Scale bars are 15 μm. I. WT-Jacob-GFP associates with the nuclear rim and this association is abolished upon the treatment with LMB (0.1 nM). The schematic drawing indicates the timeline of the time-lapse experiment. Scale bar indicates 10 μm. **j**, **k** Confocal images of hippocampal primary neurons (DIV16/18) treated with 0.1 nM LMB showing enhanced nuclear IR of endogenous Jacob. Images represented in color code look-up-table (Jacob LUT) show an uneven nuclear distribution. Cultures were fixed 30 min after treatment. The region of interest for quantification was identified based on a DAPI staining. (***) indicate *p* < 0.001 (ANOVA, Bonferroni). Scale bar indicates 10 μm

We next asked if Jacob will associate with the INM following nuclear import, or if binding to LaminB1 is related to nuclear export. Screening of the primary structure of Jacob revealed a candidate sequence for a Class 1a leucine-rich nuclear export signal (NES, amino acid 476-485, LFQNLRTLMT / Fig. 3a [37]). To test the functionality of this NES, we introduced a point mutation of the Ф_1_ (L476A), diminishing the number of hydrophobic consensus residues within the specific motif, and found that mutant protein (Jacob-NES-L476A-GFP) is preferentially retained in the nucleoplasm following expression in hippocampal neurons (Fig. 3a, b). However, the accumulation at the nuclear rim was no longer visible (Fig. 3b). This surprising finding then prompted us to investigate whether Jacob indeed associates with the INM via interaction with CRM1, an evolutionarily conserved receptor for the nuclear export signal of proteins [38]. To address this question, we performed a pull-down assay with recombinant Jacob and a HEK293T cell lysate containing CRM1. The pull-down assay confirmed an association of Jacob with the nuclear export receptor (Fig. 3c). No interaction was seen with the Ф_1_ (L476A) mutant of Jacob (Fig. 3d) in a pull-down assay performed with recombinant CRM1.

It has been shown that the binding of a leucine-rich NES in cargo proteins to CRM1 is stabilized by active RanGTP [39, 40]. Therefore, we next co-expressed either WT-Jacob-GFP or Jacob with the mutant NES together with either a mCherry-tagged GTPase deficient form of Ran (pmCherry-RanQ69L, [41, 42]) or with a GDP-locked form of Ran (RanT24N-mCgerry, [27, 43]). Wild-type Jacob fused to GFP when expressed alone showed a clear association with the nuclear envelope (Fig. 3e, Additional file 2: Fig. S1). Expression of the RanGTP-locked form of Ran, which favours association with CRM1, resulted in a cytosolic distribution of Jacob (Fig. 3f, Additional file 2: Fig. S1). Interestingly, the cytosolic distribution was abolished when a point mutation was introduced within Jacob’s NES. Also, the association of mNES-Jacob with the nuclear rim was decreased (Fig. 3g, Additional file 2: Fig. S1). However, Jacob remained in the nucleus and was found in close association with the nuclear envelope when co-expressed with the GDP-bound form of Ran (Fig. 3h). These findings confirm the involvement of a classical CRM1-RanGTP-dependent mechanism for both the nuclear export of Jacob and the association with the nuclear envelope.

Given that Jacob harbours a functional nuclear export sequence that is responsible for the association with the INM, and that Jacob seems to be actively exported from the nucleus via a CRM1- and RanGTP-dependent mechanism, we decided to explore whether interruption of this process will also affect the INM localization of Jacob. Leptomycin B (LMB) is an inhibitor of nuclear export that can be used to study nucleo-cytoplasmic translocation. The cellular target of LMB is CRM1 [44]. Treatment with LMB resulted in the dissociation of Jacob from the nuclear envelope and its nucleoplasmic retention (Fig. 3i) as evidenced by time-lapse imaging (Additional file 3: movie S2) of a WT-Jacob-GFP fusion protein and immunocytochemical detection of the endogenous protein (Fig. 3j, k). This indicates the involvement of CRM1 in both the association with the INM and nuclear export of Jacob.

## Discussion

**Fig. 4:**
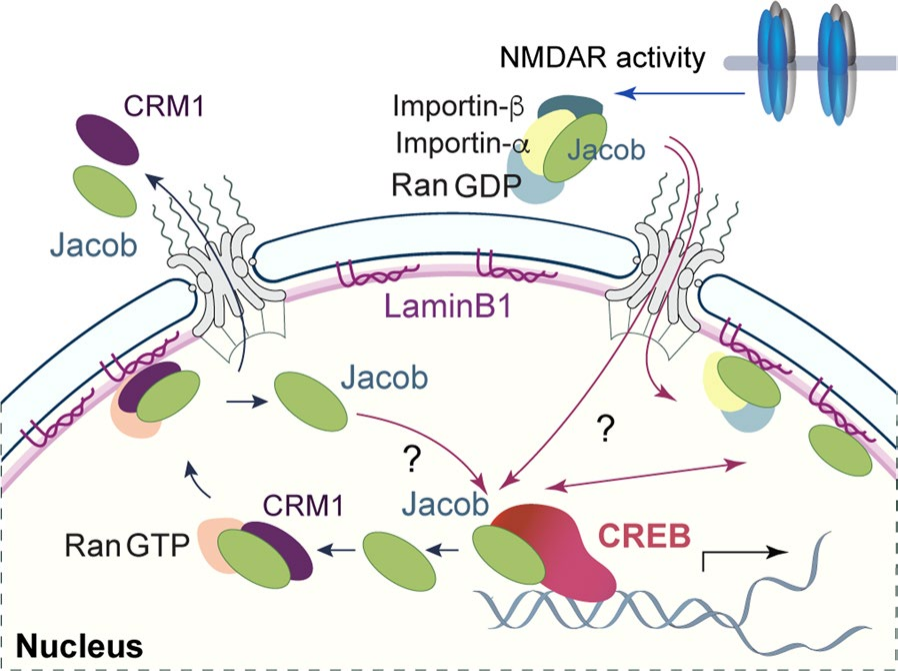
Basal NMDAR activity drives the synapto-nuclear messenger protein Jacob in NLS-dependent manner into the nucleus where it transiently but prominently localizes to the nuclear envelope via interaction with LaminB1. Association with the nuclear periphery is also mediated by a NES-CRM1-RanGTP dependent mechanism

In previous work, we have found that GluN2B-containing NMDAR are instrumental for long-distance transport and nuclear import of Jacob [14, 16, 45]. Chronic blockade of these receptors therefore depletes nuclear Jacob. Protein transport from synapse to nucleus is a complex and multi-step process [46–48]. Transport processes have been studied to a certain degree, yet many questions remain. Very little is known about the localization and the dynamics of redistribution between nuclear substructures of such proteins following nuclear entry. Neuronal nuclei are highly compartmentalized structures with defined functional subdomains [49]. In this study, we revealed an association of Jacob at the INM that is regulated by both NMDAR activity-dependent nuclear import and also nuclear export of the protein (Fig. 4). The fact that Jacob, lacking the NLS, neither entered the nucleus nor localized to the nuclear envelope, prompted us to provide direct evidence for the association with the INM, by using immunogold EM and two-color STED microscopy. The localization of Jacob at the INM is likely mediated by a direct interaction with LaminB1 which was confirmed by PLA and STED microscopy (Fig. 2l–n). At present, we cannot exclude the possibility that Jacob will also bind to LaminB2 and LaminA/C, which are similar to LaminB1. Moreover, we identified a nuclear export signal in Jacob that is bound by CRM1. Collectively, the data suggest that association of Jacob to LaminB1 is linked to both the nuclear import and the nuclear export machinery (Fig. 4). The pool of nuclear Jacob that will localize at the INM is dynamic in the sense that activity of GluN2B-containing NMDARs drives nuclear import and that no stable pool seems to reside at the INM (Fig. 4). The question therefore arises whether Jacob at the INM fulfills a nuclear function, or whether this localization is only indicative for the transit of the protein out of the nucleus.

The nuclear lamina is a filamentous protein meshwork at the interface of chromatin and the nuclear membrane [1, 2]. Apart from docking Jacob to the INM, the direct interaction with LaminB1 could also have other functional implications. Lamins are involved in invaginations of the nuclear membrane that directly affect chromatin organization. In most cases, this results in gene silencing. However, recent work points to activity-dependent alterations in genome topology and the re-localization of gene loci to “transcription factories” that are in proximity to the INM [5, 50]. Along these lines, lamins function as a scaffold for binding of transcription factors [1, 2, 6, 9–11]. At present, it is unclear whether plasticity-related gene expression regulated by nuclear Jacob will occur in such factories. However, the association of Jacob with CREB is not limited to membrane-proximal regions, [20] and we cannot exclude the possibility of a role of the protein in the opposite process, i.e. gene silencing.

Alternatively, it is conceivable that the nuclear lamina merely provides a docking site for an intermediate step relevant for nuclear export of Jacob. Our data support two non-mutually exclusive scenarios. Since the interaction of Jacob with the nuclear lamina is direct, the association of CRM1 with the NES should be dispensable for binding of Jacob to LaminB1. It is, however, puzzling that in a cellular context the presence of the NES is required for the localization at the INM and that LMB results in dissociation of the overexpressed protein from the nuclear lamina. Thus, CRM1-binding to Jacob appears to promote the localization at the INM as an intermediate step before nuclear export. In this scenario, another factor would be required to actively release the complex from the nuclear lamina for nuclear export. On the other hand, it is possible that re-modeling of the Jacob complex is a prerequisite for nuclear export. Activity-dependent nucleocytoplasmic shuttling has been shown for many regulators of transcription and, in this context, nuclear residing time is a crucial factor, as is protein degradation. Processes that eventually contribute to the destiny of Jacob following nuclear export are likely regulated and will occur at the nuclear lamina. Finally, we show here for the first time that synapto-nuclear messenger proteins display transient localization and association with the nuclear periphery. The question arises whether this is a feature specific to Jacob. The current study paves the way to address these questions and further research is needed to explore the underlying molecular mechanisms.

## Conclusion

We propose that the nuclear function of the synapto-nuclear messenger protein Jacob relies on redistribution processes that will likely occur in a stepwise manner. A transient association with the inner nuclear membrane is regulated by multiple factors: NMDAR activity and nuclear import, direct interaction with LaminB1, and NES-CRM1-dependent mechanisms.

## Supplementary Information

**Additional file 1: Movie S1.**

**Additional file 2: Figure S1.** The RanGTP-CRM1-dependent mechanism is critical for Jacob`s subcellular distribution and association with the nuclear rim. A. Association of WT-Jacob-GFP with the nuclear rim was quantified based on LaminB1 stainings. The threshold was applied to LaminB1 image, the ring was interrupted (*) and ROIs were outlined using the wand tool (Fiji; indicated with the arrow). Laser settings and signal amplification parameters throughout the image acquisition of overexpressed Jacob were kept constant. Only those cells that fitted into the dynamic range were selected for quantification. Scale bar indicates 10 μm. B. Graph represents relative amounts of WT-Jacob/mNES-Jacob at the nuclear rim when co-expressed either with RanQ96L or RanT24N normalized to control (mCherry).

**Additional file 3: Movie S2.**

## Data Availability

The datasets used and/or analyzed during the current study available from the corresponding author on reasonable request.

## Abbreviations

APV: D(-)-2-amino-5-phosphonopentanoic acid
BSA: Bovine serum albumin
CRM1: Chromosomal maintenance 1, also known as Exportin 1
CREB: CAMP response element binding protein
DAPI: 4′,6-Diamidin-2-phenylindol
DTT: Dithiothreitol
EDTA: Ethylenediaminetetraacetate
EM: Electron microscopy
ER: Endoplasmatic reticulum
GFP: Green fluorescent protein
GluN2B: Glutamate NMDA receptor subunit epsilon-2
INM: Inner nuclear membrane
IPTG: Isopropyl-β-d-thiogalactopyranoside
LMB: Leptomycin B
NES: Nuclear export signal
NGS: Normal goat serum
NLS: Nuclear localization signal
NMDAR: *N*-Methyl-d-aspartate receptors
NVP-AAM077: [[[(1S)-1-(4-Bromophenyl)ethyl]amino](1,2,3,4-tetrahydro-2,3-dioxo-5-quinoxalinyl)methyl] phosphonic acid tetrasodium salt
ONM: Outer nuclear membrane
PBS: Phosphate-buffered saline
PFA: Paraformaldehyde
RanGDP/GTP: Guanosine-5′-triphosphate bound Ras-related nuclear protein in GDP/GTP bound form
SDS: Sodium dodecyl sulfate
RFP: Red fluorescent protein
