# Supplementary figures and images for: The nuclear lamina is a hub for the nuclear function of Jacob
Source: Mol Brain. 2021 Jan 12;14:9. doi: 10.1186/s13041-020-00722-1 (PMC7802242; doi:10.1186/s13041-020-00722-1)

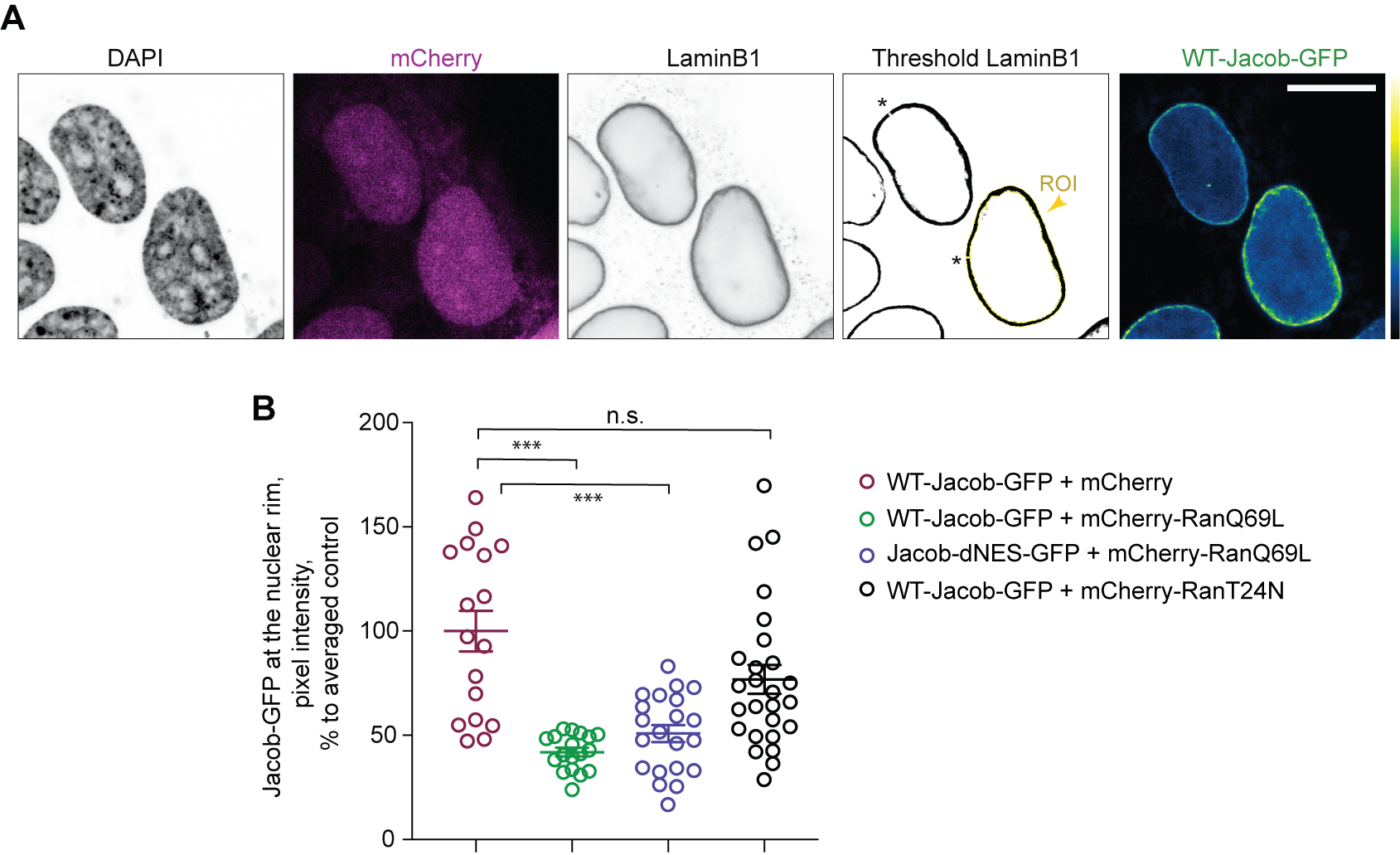

Supplement: Supplementary file 2 — Additional file 2: Figure S1. The RanGTP-CRM1-dependent mechanism is critical for Jacob`s subcellular distribution and association with the nuclear rim. A. Association of WT-Jacob-GFP with the nuclear rim was quantified based on LaminB1 stainings. The threshold was applied to LaminB1 image, the ring was interrupted (*) and ROIs were outlined using the wand tool (Fiji; indicated with the arrow). Laser settings and signal amplification parameters throughout the image acquisition of overexpressed Jacob were kept constant. Only those cells that fitted into the dynamic range were selected for quantification. Scale bar indicates 10 μm. B. Graph represents relative amounts of WT-Jacob/mNES-Jacob at the nuclear rim when co-expressed either with RanQ96L or RanT24N normalized to control (mCherry). [file 13041_2020_722_MOESM2_ESM.tif]
